# Association between Submucosal Fibrosis and Endoscopic Submucosal Dissection of Recurrent Esophageal Squamous Cell Cancers after Chemoradiotherapy

**DOI:** 10.3390/cancers14194685

**Published:** 2022-09-26

**Authors:** Tsunetaka Kato, Takuto Hikichi, Jun Nakamura, Minami Hashimoto, Ryoichiro Kobashi, Takumi Yanagita, Rei Suzuki, Mitsuru Sugimoto, Yuki Sato, Hiroki Irie, Mika Takasumi, Yuka Oka, Tadayuki Takagi, Yuko Hashimoto, Masao Kobayakawa, Hiromasa Ohira

**Affiliations:** 1 Department of Endoscopy, Fukushima Medical University Hospital, Fukushima 960-1295, Japan; 2 Department of Gastroenterology, Fukushima Medical University School of Medicine, Fukushima 960-1295, Japan; 3 Department of Diagnostic Pathology, Fukushima Medical University School of Medicine, Fukushima 960-1295, Japan; 4 Medical Research Center, Fukushima Medical University, Fukushima 960-1295, Japan

**Keywords:** chemoradiotherapy, endoscopic submucosal dissection, esophageal cancer, radiation, recurrence

## Abstract

**Simple Summary:**

The efficacy and safety of endoscopic submucosal dissection for early esophageal cancer after chemoradiotherapy have not been established. In this study, we focused on the fibrosis of the submucosa. As a result, we found that endoscopic submucosal dissection for early esophageal cancer can be performed reliably without adverse events, but the procedure takes longer for lesions with strong fibrosis of the submucosa.

**Abstract:**

Endoscopic resection is a treatment of choice for a metachronous early-stage esophageal squamous cell carcinoma (ESCC) appearing after a radical cure of esophageal cancer by chemoradiotherapy (CRT). However, non-curative resection, and procedural complications including perforation due to radiation-induced submucosal fibrosis, are a concern. This study aimed to evaluate the association between submucosal fibrosis and the usefulness and safety of endoscopic submucosal dissection (ESD) in ESCC after CRT. This study retrospectively analyzed 13 lesions in 11 patients in our institute. Submucosal fibrosis under the lesion (F score) was classified into three levels (F0: none or mild, F1: moderate, and F2: severe) based on endoscopic and histopathologic findings. All lesions were F1 or greater (F1: 8 lesions and F2: 5 lesions). En bloc and R0 resection rates were both 100%. The procedural speed was slower in F2 than in F1 (F1 vs. F2; 15.1 mm^2^/min vs. 7.1 mm^2^/min, *p* = 0.019), without procedure-related adverse events. At a median follow-up of 42 months (range: 14–117 months) after ESD, 7 of 11 (63.6%) patients were alive without recurrence, and without ESCC-related death. ESCC after CRT reliably and safely resected en bloc by ESD but was more difficult in lesions with strong submucosal fibrosis.

## 1. Introduction

Chemoradiotherapy (CRT) has been used to treat various esophageal cancer stages because of its potential for complete response (CR) and esophageal preservation [1,2,3,4,5,6,7,8]. The CR rate of CRT for esophageal cancer was 87.5% for clinical Stage I [2] and 62.2% for clinical Stages II–III [9].

Squamous cell carcinoma (SCC) is a significant histologic type of esophageal cancer in Japan and other Asian countries, with 12.2–29% of patients having multiple SCCs in the esophagus, either synchronously or metachronously [10,11,12,13,14]. Recently, an increasing number of patients with esophageal SCC (ESCC) who survive long-term and new ESCC metachronously have appeared during the follow-up. Endoscopic resection (ER) is a treatment for metachronous recurrent early-stage ESCC after CRT, and the usefulness of endoscopic submucosal dissection (ESD), which can obtain a reliable en bloc resection, was reported [15,16,17,18,19,20,21,22,23]. Resecting en bloc or treating with ER for ESCC after CRT may be difficult due to submucosal fibrosis [9,15,24], and the ESD efficacy was not adequately evaluated.

This study aimed to assess the association between submucosal fibrosis and the outcomes and safety of ESD in patients with ESCC with a history of CRT for esophageal cancer.

## 2. Materials and Methods

### 2.1. Patients

This study included patients with ESCC who underwent ESD at Fukushima Medical University Hospital from January 2012 to June 2021. The inclusive criteria were as follows: (1) patients who had a history of CRT for esophageal cancer, (2) patients who were diagnosed with intramucosal esophageal cancer before ESD [25], (3) patients whose cancer was considered to develop within irradiation area, (4) patients whose medical records showed detailed information during CRT. Patients who received special treatment other than conventional radiotherapies, such as proton therapy, were excluded from the study.

This study complied with the standards of the Declaration of Helsinki and current ethics guidelines. This retrospective study was conducted with the approval of the Ethics Committee of Fukushima Medical University (approval No. 2020-146).

### 2.2. ESD Procedure and Pathological Evaluation

ESD was performed under the general anesthesiologist’s control. All ESDs were performed by expert physicians who had performed > 50 esophageal ESDs, or by non-expert physicians under the supervision of expert physicians, according to previous reports [26,27,28,29,30,31,32]. 

An endoscope (GIF-Q260J or GIF-H290T; Olympus Medical Systems Corp., Tokyo, Japan) was used with carbon dioxide, and A VIO300D or VIO3 (ERBE Elektromedizin, Tübingen, Germany) was used as the high-frequency system. The area around the lesion was marked using a Dual knife (Olympus Medical Systems Corp.), which was then used to incise the mucosa after injecting 0.4% sodium hyaluronate (MucoUp; Boston Scientific Japan, Tokyo, Japan) into the submucosa using a needle (ImpactFlow; TOP Corp., Tokyo, Japan) [33,34,35]. An IT knife nano (Olympus Medical Systems Corp.) was mainly used for submucosal dissection, and SB knife Jr (SB Kawasumi, Co., Ltd., Tokyo, Japan), a scissors-type knife, was used in cases of severe submucosal fibrosis. Furthermore, coagulation was performed using hemostatic forceps (Coagrasper; Olympus Medical Systems Corp.) for bleeding and hemorrhage prevention. 

Triamcinolone is injected into the mucosal defect immediately after resecting the lesion to prevent postoperative stenosis if the circumference of the mucosal defect after resection was more than 3/4 circumference [36,37,38].

### 2.3. Histopathological Assessment in the Resected Specimen of ESD

Pathologists (Y.O., Y.H.) evaluated lesion size, histological type, cancer depth, macroscopic type, lymphovascular invasion (LVI) such as lymphatic invasion (Ly) and venous invasion (V), horizontal margin (HM), and vertical margin (VM) based on the Japanese classification of esophageal cancer [25]. LVI was assessed by hematoxylin and eosin-staining (HE-staining) and D2-40 staining [39], and Elastica Masson staining was used to assess submucosal fibrosis.

### 2.4. Outcomes

The en bloc resection rate, R0 resection rate, procedural speed, procedure-related adverse events, and prognosis were evaluated. The procedural speed (mm^2^/min) was calculated by dividing the dissection area (mm^2^) by the procedure time (min). The procedure time was defined as the time from the start of the mucosal incision to the end of lesion resection, and the dissection area was calculated as the radius of the long axis × radius of the short axis × 3.14.

Regarding procedure-related adverse events, perforation was defined as endoscopic confirmation of the mediastinum or free air on computed tomography (CT). CT was performed only when endoscopically suspected perforation was detected during ESD. Postoperative bleeding was defined as the presence of hematemesis or black stools after ESD and active bleeding or exposed blood vessels on endoscopy [40].

**Figure 1:**
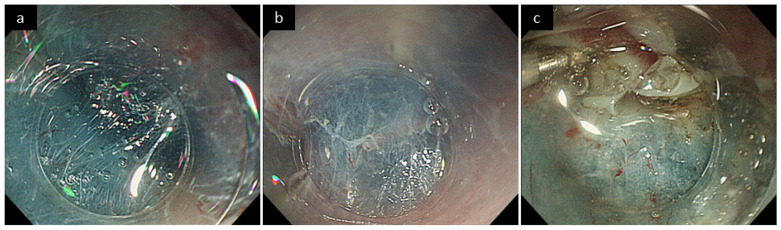
Evaluation criteria for endoscopic F score. (**a**) None or mild fibrosis (eF0). The submucosa is not whitish, and submucosa is sufficiently elevated by local injection. (**b**) Moderate fibrosis e(F1). The submucosa looks slightly white, but the submucosal elevation is possible. (**c**) Severe fibrosis (eF2). The submucosa looks highly white, and submucosal elevation is inadequate.

**Figure 2:**
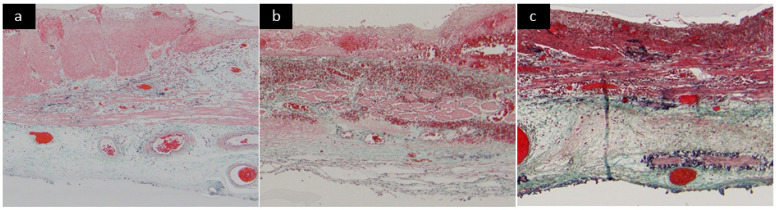
Evaluation criteria for pathological F score. (**a**) None or mild fibrosis (pF0). Fibrosis was defined as mild when fibrous tissue was <20%. (**b**) Moderate fibrosis (pF1). Fibrosis was defined as moderate when fibrous tissue was between 20% and 60%. (**c**) Severe fibrosis (pF2). Fibrosis was defined as severe when fibrous tissue was 60% or more.

**Figure 3:**
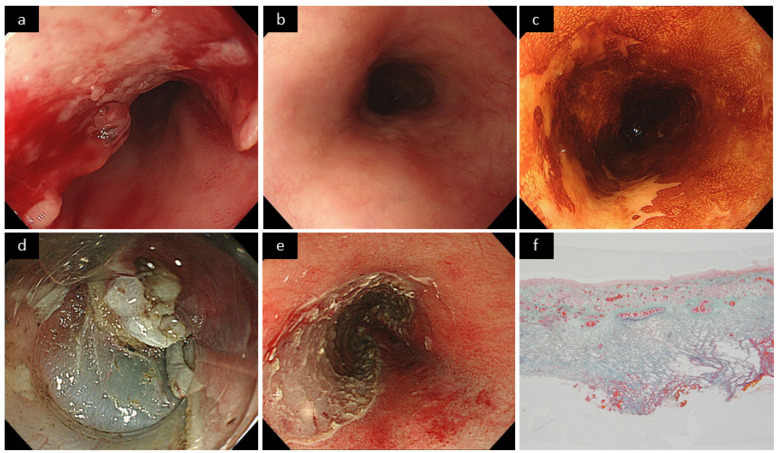
A representative case (Case 10). (**a**) An advanced squamous cell carcinoma (SCC) in the middle thoracic esophagus was seen. (**b**) After definitive chemoradiotherapy (dCRT), no tumor remnant was observed and a complete response was obtained. (**c**) Four years after the dCRT, recurrence of half-circumscribed superficial esophageal SCC in the upper thoracic esophagus. (**d**) During endoscopic submucosal dissection (ESD), severe submucosal fibrosis was seen. The endoscopic F score was evaluated as 2. (**e**) ESD was completed without adverse events. As the mucosal defect was 3/4 circumference, a triamcinolone injection was performed to prevent stenosis. (**f**) Histopathological evaluation of the resection specimen showed severe submucosal fibrosis, with a pathological fibrosis score of 2.

Submucosal fibrosis under the lesion was named as “F score.” The F score based on endoscopic and pathological findings was defined as the endoscopic F (eF) score and pathological F (pF) score, respectively. The eF score was evaluated by two endoscopists (T.K. and R.K.) based on the stored endoscopic images and videos and was assigned to 0 (none or mild fibrosis), 1 (moderate fibrosis), and 2 (severe fibrosis) by agreement between the two endoscopists (Figure 1) [41]. The pF score was evaluated by one pathologist (Y.O.) as 0 (none or mild fibrosis), 1 (moderate fibrosis), and 2 (severe fibrosis) based on the Elastica Masson staining of the ESD specimen (Figure 2) [15]. Finally, the higher score between eF and pF scores was defined as the F score of the lesion (Figure 3).

Regarding the patient characteristics, the disease stage before CRT in patients undergoing definitive CRT (dCRT) was evaluated based on esophagogastroduodenoscopy (EGD), endoscopic ultrasound, CT, and positron emission tomography (PET). Patients who underwent CRT as an additional treatment after ESD were evaluated based on the histopathological results of ESD, CT, and PET. Patients were followed up with EGD and CT once or twice a year after ESD.

### 2.5. Statistical Analysis

All identified patients were analyzed, and subgroup analyses by submucosal fibrosis were conducted. Values are reported as medians with ranges. Statistically significant differences between patient characteristics and ESD results were assessed using the Mann-Whitney U test for continuous variables. Differences were significant at *p*-values of <0.05. This analysis was performed using the Statistical Package for the Social Sciences software (version 21 for windows; IBM Corp., Armonk, NY, USA).

## 3. Results

### 3.1. Patients and Lesion Characteristics

This study analyzed 13 lesions in 11 patients (Table 1). CRT was for dCRT in 8 cases and additional therapy after ESD in 3 cases, with a median total radiation dose of 60 Gy in a median follow-up of 38 months (range: 13–85 months) from the end of CRT to ESD. There were no cases of residual recurrence after CRT, and all ESCCs that underwent ESD were new lesions with metachronous recurrence.

### 3.2. Treatment Outcomes of ESD

The cancer depth was 4 (30.8%) with the epithelium, 7 (53.8%) with the lesions on the lamina propria mucosae, and 2 (15.4%) with muscularis mucosae. HM, VM, and LVI were negative in all lesions. Both en bloc resection rate and R0 resection rate were 100%. Five lesions with mucosal defect circumference of more than 3/4 after ESD were treated with triamcinolone injection and no stenosis occurred. No other adverse events, such as perforation or postoperative bleeding occurred (Table 1).

F score was determined as F1 or higher in all cases (Table 1). However, the interval between CRT and ESD (F1 vs. F2; 38 months vs. 48 months, *p* = 0.883) and RT dose (F1 vs. F2; 60 Gy vs. 60 Gy, *p* = 0.107) were not statistically significantly different between F1 and F2 (Table 2). Procedural speed was slower in F2 than in F1 (F1 vs. F2; 15.1 mm^2^/min vs. 7.1 mm^2^/min, *p* = 0.019). High-frequency knives for ESD in F2 (Lesion 7) included 3 devices (dual knife, IT knife nano, and SB knife Jr), but without difference between F1 and F2 (F1 vs. F2; 2 vs. 2, *p* = 0.558).

### 3.3. Prognosis

**Figure 4:**
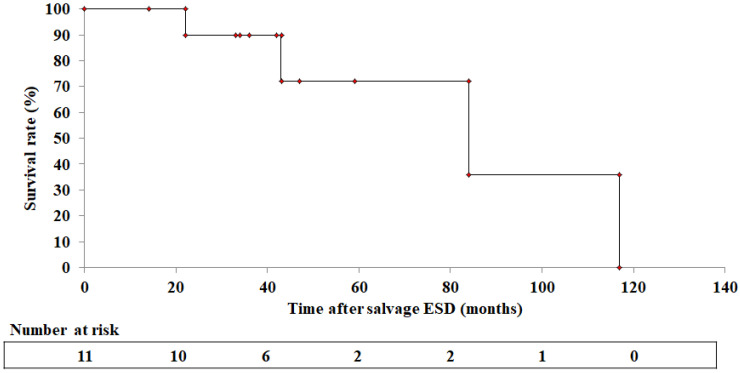
Overall survival of all 11 patients from the initiation of endoscopic submucosal dissection.

At a median follow-up of 42 months (range: 14–117 months) after ESD, 7 of 11 patients (63.6%) were alive without recurrence. There were no ESCC-related deaths, and the causes of death in the four other cases were senility, head and neck cancer, liver cirrhosis, and debilitating death due to alcoholism (Figure 4).

## 4. Discussion

This study revealed that ESD for ESCC that recurred metachronously in the irradiated area after CRT is safe and reliable for resection. Additionally, the evaluation of submucosal fibrosis under ESCC after CRT, based on endoscopic and histopathologic findings, is considered novel because the procedural speed was slow in lesions with significant fibrosis.

Surgery and photodynamic therapy (PDT) were performed for locally recurrent ESCC after CRT in esophageal cancer [42,43,44,45]. However, surgical procedures for ESCC after CRT are highly invasive with reported perioperative mortality rates as high as 7.4–25% [42,46,47,48,49,50,51]. PDT is advantageous because it treats ESCC with strong fibrosis, but its downside is that it does not allow histopathological lesion evaluation and it uses special and expensive equipment and drugs, thereby limiting the number of capable facilities [45,52,53,54,55,56]. For these treatments, ER is superior because it is minimally invasive and allows detailed pathological evaluation.

Regarding ER for locally recurrent ESCC after CRT, ESD was reported with an en bloc resection rate of 86–100% [15,18,19,21,22,23], higher than that of endoscopic mucosal resection (EMR) of 46–47% [17,18,20]. The en bloc resection rate in EMR in ESCC with local recurrence after CRT is low because of the difficulty of reliable snaring due to the submucosal fibrosis [15,16]. Conversely, ESD is feasible for ESCC resection after CRT if a secure mucosal incision and submucosal dissection can be performed. However, ESD is associated with a risk of perforation in lesions with strong submucosal fibrosis. Nagami et al. reported that prior CRT was an independent predictor of lower en bloc resection rate and perforation in ESD for ESCC [57]. Additionally, it has been reported that submucosal fibrosis increases with time after CRT [58]. However, this study found no significant difference in time course after CRT; moreover, no significant correlation was found using the Pearson product-moment correlation coefficient (correlation coefficient: 0.011, *p*-value: 0.97). Since only a small number of cases were considered in our study, further research is required to accumulate the association between submucosal fibrosis and duration after CRT.

In this study, all lesions were associated with submucosal fibrosis in ESCC with local recurrence in the irradiated area after CRT, but en bloc resection by ESD was possible in all cases. However, the procedure was difficult to perform in lesions with strong fibrosis, and the procedure speed was reduced, suggesting the need for careful treatment techniques. Furthermore, predicting submucosal fibrosis before ESD was difficult; therefore, ESD was performed.

Several studies on the prognosis of patients who underwent ER after CRT for esophageal cancer showed a 61.9–84.2% disease-specific survival range at 18–54 months of follow-up after ER, and a 0–46.7% all-cause death rate [16,17,19,21,22,23]. The 3-year overall survival (OS) was 56.1–75% and 41.6–49.1% for the 5-year OS [16,17,19,21,22,23] (Table 3). The present study revealed no ESCC-related deaths at a median follow-up of 42 months (range: 14–117 months) after ESD, although four all-cause deaths were observed among 11 patients. The 3-year and 5-year OS rates were 90% and 72%, respectively. Therefore, our results suggest that ESD can improve the prognosis if ESCC is detected in early-stage cancer after CRT although it may be related to the fact that all patients who underwent ESD in this study had metachronous lesions, rather than residual or local recurrence.

This study has several limitations. First, it was a single-center, retrospective study with small sample size. Second, the evaluated submucosal fibrosis from endoscopic images was evaluated from stored images and written records. Pathologic fibrosis may also be underestimated because it is limited to the presence of fibrosis in the resected specimen. Third, ESD was performed by more than one endoscopist.

## 5. Conclusions

All ESCCs with metachronous recurrence after CRT were associated with moderate or high fibrosis endoscopically or histopathologically. Furthermore, ESCC after CRT reliably and safely resected en bloc by ESD but was more difficult in lesions with strong submucosal fibrosis. Thus, further prospective multicenter studies are needed to establish new evidence.

## Figures and Tables

**Table 1:** Patient characteristics and ESD results.

Lesion	Age	T Stage before CRT	CRT	Interval from CRT to ESD, m	Location	Patterns of Recurrence	Procedure Time, min	Lesion Size, mm	Circumference of Mucosal Defect	F Score (eF/pF)	R0 Resection	Histological Type	Cancer Depth
Case 1-1	75	T4	dCRT	26	Ce	Metachronous	43	41 × 37	3/5	1 (1/1)	Yes	SCC	MM
Case 1-2	75	T4	dCRT	26	Ut	Metachronous	20	24 × 17	1/3	1 (1/1)	Yes	SCC	LPM
Case 2-1	78	T1a	aCRT	37	Mt	Metachronous	50	40 × 22	7/8	1 (1/1)	Yes	SCC	LPM
Case 2-2	79	T1a	aCRT	49	Ut	Metachronous	40	30 × 24	1/3	1 (1/0)	Yes	SCC	LPM
Case 3	55	T1b	aCRT *	48	Mt	Metachronous	30	21 × 13	1/3	2 (2/1)	Yes	SCC	EP
Case 4	88	T1b	dCRT	63	Mt	Metachronous	75	35 × 25	1/2	1 (1/0)	Yes	SCC	EP
Case 5	75	T4	dCRT	19	Mt	Metachronous	72	34 × 24	1/2	2 (2/1)	Yes	SCC	MM
Case 6	67	T4	dCRT	85	Mt	Metachronous	32	32 × 24	3/4	1 (1/1)	Yes	SCC	LPM
Case 7	76	T1a	aCRT	24	Mt	Metachronous	57	23 × 4	1/2	2 (2/1)	Yes	SCC	EP
Case 8	64	T3	dCRT	74	Mt	Metachronous	47	27 × 23	1/4	2 (2/1)	Yes	SCC	LPM
Case 9	84	T3	dCRT	13	Mt	Metachronous	57	32 × 16	3/4	1 (1/1)	Yes	SCC	LPM
Case 10	66	T4	dCRT	48	Ut	Metachronous	221	30 × 25	4/5	2 (2/2)	Yes	SCC	EP
Case 11	77	T1b	dCRT	38	Lt	Metachronous	90	56 × 42	7/8	1 (0/1)	Yes	SCC	LPM

ESD: endoscopic submucosal dissection, CRT: chemoradiation therapy, F score: submucosal fibrosis score, eF: endoscopic fibrosis score, pF score: pathological fibrosis score, dCRT: definitive chemoradiotherapy, aCRT: additional therapy after ESD Ce: cervical esophagus, Ut: upper thoracic esophagus, Mt: middle thoracic esophagus, Lt: lower thoracic esophagus. SCC: squamous cell carcinoma, EP: epithelium, LPM: lamina propria mucosae, MM: muscularis mucosae. Case 1-1 and case 1-2 were the recurrent lesions identified 26 months after CRT in the same patient, both of which were treated on the same day. Case 2-1 and case 2-2 were recurrent lesions found in the same patient and treated 37 months and 49 months after CRT. * Radiation was discontinued at up to 40 Gy due to adverse events.

**Table 2:** Relationship between ESD results and Fibrosis scores.

Evaluation Items	Total (*n* = 13)	F1 (*n* = 8)	F2 *(n* = 5)	*p* Value
Lesion size (mm), median (range)	21 (4–31)	21.5 (4–30)	14 (6–31)	0.661 *
Resected specimen size (mm), median (range)	32 (21–56)	33.5 (24–56)	27 (21–34)	0.048 *
En bloc resection rate, % (n)	100 (13)	100 (8)	100 (5)	-
R0 resection rate, % (*n*)	100 (13)	100 (8)	100 (5)	-
Interval between CRT and ESD (month), median (range)	38 (13–85)	38 (13–85)	48 (19–74)	0.883 *
Radiation dose (Gy), median (range)	60 (40–85)	60 (60–70)	60 (40–85)	0.107 *
Procedure speed (mm^2^/min), median (range)	10.4 (2.7–27.7)	15.1 (7.1–27.7)	7.1 (2.7–10.4)	0.019 *
Number of high-frequency knives used, median (range)	2 (1–3)	2 (2–2)	2 (1–3)	0.558 *
Procedure-related adverse events, *n*	0	0	0	-

* *p*-values were calculated using the Mann–Whitney U test. ESD: endoscopic submucosal dissection, CRT: chemoradiation therapy. Procedure speed (mm^2^/min); dissection area (radius of long axis × radius of short axis × 3.14 (mm^2^)/procedure time (min).

**Table 3:** Literature reports regarding endoscopic resection of recurrent cases with ESCC after chemoradiotherapy.

First Author	Number of Patients (Type of Recurrence)	Treatment	En Bloc Resection Rate, %	R0 Resection Rate, %	Adverse Events, n	Follow-Up Period (Months)	Death of ESCC, n	Death of Other Diseases, %	Disease-Specific Survival Rate, %	3-Year OS, %	5-Year OS, %
Yano et al. [16]	21, (local recurrence: 13, local remnant: 8)	EMR	33 (7/21)	N/A	None	54	8	14.3 (3/21)	61.9 (13/21)	56.1	49.1
Makazu et al. [17]	11 (13 lesions), (local recurrence: 9, local remnant: 2)	EMR	46.2 (6/13)	84.6 (11/13)	None	38.9	4	18.2 (2/11)	63.6 (7/11)	62.3	41.6
Takeuchi et al. [19]	19, (local recurrence: 15, local remnant: 4)	ESD	100 (19/19)	94.7 (18/19)	None	54.6	3	31.6 (6/19)	84.2 (16/19)	74	N/A
Koizumi et al. [21]	12 (local recurrences)	ESD	91.7 (11/12)	91.7 (11/12)	2 (stenosis)	18	3	0 (0/12)	75 (9/12)	N/A	N/A
Nakajo et al. [22]	33 (35 lesions), (local recurrences or remnant)	ESD	85.7 (30/35)	N/A	None	18	0	N/A	N/A 1-year: 100	N/A 1-year: 95.8	N/A
Nakajo et al. [22]	25 (34 lesions), (metachronous)	ESD	100 (34/34)	N/A	None	19	1	N/A	N/A 1-year: 94.1	N/A 1-year: 94.1	N/A
Kimura et al. [23]	30 (33 lesions), (local recurrence: 27, local remnant: 6)	ESD	94 (31/33)	57.6 (19/33)	7 (stenosis) 1 (perforation)	51	7	46.7 (14/30)	76.7 (23/30)	75	N/A
Our study	11 (13 lesions), (metachronous)	ESD	100 (13/13)	100 (13/13)	None	42	0	36.4 (4/11)	100 (11/11)	90	72

ESCC: esophageal squamous cell carcinoma, OS: overall survival, EMR: endoscopic mucosal resection, ESD: endoscopic submucosal dissection, N/A: not available.

## Data Availability

Data available on request due to restrictions, e.g., privacy or ethical.
